# Exosomal delivery of METTL3 promotes M1 macrophage polarization by inducing miR-155-5p maturation via m6A modification

**DOI:** 10.1080/07853890.2026.2708378

**Published:** 2026-07-31

**Authors:** Xinyu Wu, Hongbo Gong, Jie Zhang, Ling Yu, Xiangyu Yan, Wanlong Pan, Chaoyue Liu

**Affiliations:** ^a^ Basic Medical Experimental Teaching Center, Institute of Basic Medicine, North Sichuan Medical College, Sichuan, Nanchong, China; ^b^ The Second People’s Hospital of Nanchong, Sichuan, Nanchong, China

**Keywords:** Exosomes, N6-methyladenosine modification, METTL3, miR-155-5p, macrophage polarization

## Abstract

**Objective:**

Macrophage polarization and function are regulated by epigenetic mechanisms and microRNAs transported by exosomes; however, the specific mechanisms are not clear yet. The study aims to investigate whether exosomes regulate miR-155-5p maturation through methyltransferase-like 3 (METTL3)-mediated N6-methyladenosine (m6A) modification, thereby driving M1 macrophage polarization and providing potential targets for inflammation and tumor immunotherapy.

**Method:**

THP-1 cells were differentiated into M0 macrophages by PMA and subsequently polarized to the M1 phenotype with LPS and IFN-γ. Exosomes were extracted from culture supernatants using ultracentrifugation, and identified by dynamic light scattering (DLS), transmission electron microscopy (TEM), and western blotting (WB). Genes for overexpression or knockdown of METTL3/miR-155-5p were loaded into M0 exosomes, respectively, and M0-exos were then used to deliver them to recipient macrophages or injected into mice.

**Results:**

Dot blot analysis showed an increase in m6A modification levels in the OE-METTL3 group. Flow cytometry (FCM), Enzyme-linked immunosorbent assay (ELISA), RT-qPCR, and WB results indicated that the OE-METTL3 group exhibited significantly elevated expression of M1 macrophage markers CD86, iNOS and HLA-DR, cytokines IL-1β, IL-6, TNF-α, and IL-18, signaling molecules NLRP3, TLR4, and caspase-1, whereas the expression of M2 macrophage markers CD206, CD163, Arg-1, IL-10 and TGF-β was decreased. The SI-METTL3 group showed the opposite trends. Further results demonstrated that miR-155-5p was correspondingly upregulated or downregulated. Additional intervention with miR-155-5p confirmed its regulatory effect on m6A modification and M1 polarization (*p* < 0.05). *In vivo* experiments yielded consistent results.

**Conclusion:**

Exosomes promote miR-155-5p maturation through METTL3-mediated m6A modification, activate the NLRP3/TLR4/caspase-1 signaling pathway, drive M1 macrophage polarization, and exacerbate inflammatory responses. This research may provide novel potential targets for inflammation and tumor immunotherapy.

## 1. Introduction

Macrophages, as the core effector cells of the innate immunity system, perform fundamental functions including phagocytosis, sterilization, and antigen presentation. They also serve as a key bridge connecting innate and adaptive immunity [1]. High plasticity, a key characteristic of macrophages, enables them to differentiate into distinct functional phenotypes in response to microenvironmental signals [2].

M1 macrophages typically exhibit high expression of surface markers such as iNOS and CD80/CD86, and secrete substantial amounts of pro-inflammatory cytokines including tumour necrosis factor-α (TNF-α), interleukin-1β (IL-1β) and interleukin-6 (IL-6). Their polarization is precisely regulated by key signalling molecules and complexes, such as the nucleotide-binding oligomerization domain-like receptor protein 3 (NLRP3) inflammasome, Toll-like receptor 4 (TLR4), and caspase-1 [3–4]. These players are central to host defence against pathogens, anti-tumour immunity, and responses to early tissue injury [5–7]. On the contrary, M2 macrophages highly express markers like CD163 and CD206 and secrete factors such as interleukin-10 (IL-10) and transforming growth factor-beta (TGF-β), which are mainly involved in tissue repair, angiogenesis, and immune suppression [8–10]. Disruption of the M1/M2 balance can lead to chronic inflammatory diseases such as rheumatoid arthritis, atherosclerosis, and inflammatory bowel disease [11–13]; conversely, predominant M2 polarization is related to tumor immune evasion and fibrosis progression [14–15]. Therefore, precise regulation of macrophage polarization has become an important strategy for the treatment of inflammatory diseases and tumor immunotherapy.

Recent studies have shown that macrophage polarization is precisely regulated by epigenetic mechanisms and microRNAs (miRNAs) carried by exosomes [16–18]. Among these mechanisms, N6-methyladenosine (m6A), the most abundant internal chemical modification in eukaryotic mRNA, is a reversible post-transcriptional modification dynamically regulated by *Writer*, *Eraser*, and *Reader* proteins, and is extensively involved in RNA splicing, stability, translation efficiency, and degradation [19]. M6A also plays a critical role in immune cell function regulation [20–21]. Methyltransferase-like 3 (METTL3) serves as the core catalytic *Writer* protein of the m6A methyltransferase complex and forms a functional complex with METTL14, WTAP, and other subunits to catalyze the installation of m6A modifications [22]. The role of METTL3 in macrophage function regulation has been confirmed by multiple studies: METTL3 can enhance the stability of signal transducer and activator of transcription 1 (STAT1) mRNA through m6A modification, thereby promoting interferon-γ (IFN-γ)-induced M1 polarization [23]. Meanwhile, METTL3-mediated m6A modification can also regulate the expression of key molecules in the TLR4/NF-κB signalling pathway, enhancing lipopolysaccharide (LPS)-induced inflammatory responses [24]. In addition, METTL3 plays an important role in the polarization regulation of tumour-associated macrophages (TAMs) by affecting M2-type marker expression through m6A modification, thereby modulating the immunosuppressive state in the tumour microenvironment [25–26]. These studies have established METTL3 as a key node in macrophage function regulation.

Meanwhile, miRNAs function as important post-transcriptional regulators [27], and their biogenesis and function are also regulated by m6A modification, which in turn influences macrophage polarization [28–30]. These processes can be harnessed to construct post-transcriptional modification regulatory networks *via* exosome-mediated delivery of miRNAs [31–34]. It is worth noting that exosomes bilayer membrane vesicles (30–200 nm in diameter) can encapsulate bioactive molecules, including proteins and nucleic acids (such as miRNAs), and deliver them to recipient cells, thereby mediating intercellular communication [35–37]. Recent studies have found that exosomes, as important carriers of intercellular communication, can deliver miR-155-5p to recipient cells and regulate macrophage polarization status. In a hypervirulent Klebsiella pneumoniae-induced acute lung injury model, activated macrophage-derived exosomes are enriched with miR-155-5p, which drives extensive macrophage M1 polarization and inflammatory responses by targeting mitogen- and stress-activated protein kinase 1 (MSK1) and activating the p38/MAPK signalling pathway [38]. MiR-155 in M1 macrophage exosomes can also affect cardiomyocyte proliferation and cardiac repair by targeting the interleukin-6 receptor (IL-6R) and inhibiting the JAK/STAT3 pathway [39–40]. These findings highlight the important role of the exosomes-miRNA axis. However, a fundamental question remains unresolved: Can exosomes act as functional carriers that deliver METTL3 to regulate the maturation of miR-155-5p *via* m6A modification, thereby precisely regulating macrophage plasticity? The potential ‘exosomes-METTL3-m6A-miR-155-5p’ regulatory axis has not yet been clearly revealed.

Therefore, this study aims to systematically investigate the effects of METTL3 and miR-155-5p carried by M0 macrophage-derived exosomes on M1 polarization-related functional molecules. We will analyse whether exosomes promote miR-155-5p maturation through METTL3-mediated m6A modification, thereby driving M1 macrophage polarization. This exploration of a novel regulatory mechanism is expected to provide a new theoretical basis and potential targets for the immunotherapy for inflammatory diseases and cancer.

## 2. Materials and methods

### 2.1. Induction of M0 and M1 macrophages

Human monocyte-derived THP-1 cells (ZQXZ Bio, China) were cultured in RPMI-1640 medium (GIBCO, USA) at 37 °C in a 5% CO_2_ incubator. This medium contained 8% fetal bovine serum (SBI, USA) and 1% penicillin-streptomycin (Thermo, USA). To induce differentiation into M0 macrophages, cells were treated with 100 ng/mL phorbol 12-myristate 13-acetate (PMA) (Beyotime, China) for 48 h. Subsequently, the medium was replaced with RPMI-1640 complete medium formulated with exosome-free serum. We then polarized M0 macrophages to the M1 phenotype with 100 ng/mL LPS (Solarbio, China) and 20 ng/mL IFN-γ (Novoprotein, China) for 24 h.

### 2.2. Extraction of exosomes

Culture M0 macrophages in RPMI-1640 complete medium formulated with exosome-free serum. Collect 300 mL of cell supernatant. Centrifuge at 3000 × *g* for 30 min at 4 °C and discard the pellet. Centrifuge the resulting supernatant at 12 000 × *g* for 45 min at 4 °C and discard the new pellet. After filtration through a 0.22-µm filter (Millipore, USA), centrifugation was performed for 2 h using a high-speed centrifuge (Hitachi CP80NX, Japan) at 4 °C and 100 000 × *g*. After centrifugation, the supernatant was discarded, and the precipitate was resuspended in pre-cooled PBS. The protein concentration of exosomes was determined using a BCA assay kit (Solarbio, China), and the samples were then stored at −80 °C for subsequent use.

### 2.3. Transmission electron microscopy (TEM) analysis

Droplets of 10 μL M0- or M1-derived exosomes were applied to a carbon-coated copper grid and allowed to settle for 5 min for adsorption. Use filter paper to remove excess liquid, and add 10 μL 2% phosphotungstic acid staining solution (Solarbio, China) dropwise for negative staining of the grid for 1 min. After removing the residual stain and air-dried overnight at room temperature, the morphology of the vesicles was observed, and images were acquired using a transmission electron microscope (Hitachi HT7700, Japan).

### 2.4. Dynamic laser scattering (DLS) analysis

A 10 μL aliquot of the exosome sample was diluted 100-fold with PBS, filtered through a 0.22-μm needle filter into a cuvette, and then analysed for concentration and particle size using a Zetasizer (ZS90, UK). Use Origin (Origin, USA) software to draw the particle size distribution map.

### 2.5. Transfection of exosomes

THP-1 monocytes (4 × 10^5^ cells/mL) were plated in 6-well plates and induced to differentiate into M0 macrophages by the addition of PMA. After 48 h, prepare for transfection by replacing the medium with RPMI-1640 pure medium and starve the cells for 1 h. Prepare a 150 μL transfection system according to the Exo Fect^™^ Exosome Transfection Reagent manual (SBI, USA), comprising 10 µL Exo Fect reagent, 20 µL nucleic acid (20 pmol si/miRNA or 5 µg plasmid DNA), 70 µL PBS, and 50 µL M0-exos (approximately 1 × 10^8^ particles). After mixing all components, the mixture was incubated at 37 °C for 10 min and transferred immediately to ice. Then, 30 µL ExoQuick TC reagent was added, followed by incubation at 4 °C for 40 min and centrifugation at 14,000 rpm for 3 min. Discard the supernatant, add 300 µL PBS, and gently resuspend the pellet. This exosome suspension was added to the cells in the 6-well plate for co-culture. After 6 h, the medium was replaced with antibiotic-free RPMI-1640 medium containing 8% exosome- free FBS, 100 ng/mL LPS, and 20 ng/mL IFN-γ. Cells were cultured for an additional 48 h before collection for analysis. All RNA sequences were purchased from RiboBio and Tsingke; sequences are listed in Table 1.

**Table 1.** Sequences.

Target Name	Species	Sense Strand / Forward Primers (5′→3′)	Antisense Strand / Reverse Primers (5′→3′)
miR-155-5p mimic	H	UUAAUGCUAAUCGUGAUAGGGGUU	CCCCUAUCACGAUUAGCAUUAAUU
miR-155-5p inhibitor	H	—	AACCCCUAUCACGAUUAGCAUUAA
pri-miR-155	H	CTGTTAATGCTAATCGTGATAG	CTGTTAATGCTAATATGTGG
miR-155-5p	H	CTGTTAATGCTAATCGTGATAG	GGCAGTGATGTTGCGGT
SI-METTL3	H	CCUGCAAGUAUGUUCACUATT	UAGUGAACAUACUUGCAGGTT
β-Actin	H	CTCCATCCTGGCCTCGCTGT	GCTGTCACCTTCACCGTTCC
METTL3	H	TTGTCTCCAACCTTCCGTAGT	CCAGATCAGAGAGGTGGTGTAG
IL-1β	H	ATGATGGCTTATTACAGTGGCAA	GTCGGAGATTCGTAGCTGGA
IL-6	H	ACTCACCTCTTCAGAACGAATTG	CCATCTTTGGAAGGTTCAGGTTG
TNF-α	H	GAGGCCAAGCCCTGGTATG	CGGGCCGATTGATCTCAGC
IL-18	H	TCTTCATTGACCAAGGAAATCGG	TCCGGGGTGCATTATCTCTAC
TLR4	H	AGACCTGTCCCTGAACCCTAT	CGATGGACTTCTAAACCAGCCA
NLRP3	H	GATCTTCGCTGCGATCAACAG	CGTGCATTATCTGAACCCCAC
caspase-1	H	GAGAAACATCCAAAAGTGAGGG	GCCTTTCTTCTGGTCAGTGC
IL-10	H	GACTTTAAGGGTTACCTGGGTTG	TCACATGCGCCTTGATGTCTG
TGF-β	H	CTAATGGTGGAAACCCACAACG	TATCGCCAGGAATTGTTGCTG
CD86	H	CTGCTCATCTATACACGGTTACC	GGAAACGTCGTACAGTTCTGTG
NOS2(iNOS)	H	AGGGACAAGCCTACCCCTC	CTCATCTCCCGTCAGTTGGT
MRC1(CD206)	H	GGGTTGCTATCACTCTCTATGC	TTTCTTGTCTGTTGCCGTAGTT
Arg-1	H	TGGACAGACTAGGAATTGGCA	CCAGTCCGTCAACATCAAAACT
β-Actin	M	CGTTGACATCCGTAAAGACC	AACAGTCCGCCTAGAAGCAC
IL-1β	M	GCAACTGTTCCTGAACTCAACT	ATCTTTTGGGGTCCGTCAACT
IL-6	M	CTCCCAACAGACCTGTCTATAC	CCATTGCACAACTCTTTTCTCA
TNF-α	M	CCTGTAGCCCACGTCGTAG	GGGAGTAGACAAGGTACAACCC
IL-18	M	GCCATGTCAGAAGACTCTTGCGTC	GTACAGTGAAGTCGGCCAAAGTTGTC
TLR4	M	ATGGCATGGCTTACACCACC	GAGGCCAATTTTGTCTCCACA
NLRP3	M	ATTACCCGCCCGAGAAAGG	TCGCAGCAAAGATCCACACAG
caspase-1	M	CCCCAGGCAAGCCAAATC	TTGAGGGTCCCAGTCAGTCC
IL-10	M	GCTCTTACTGACTGGCATGAG	CGCAGCTCTAGGAGCATGTG
TGF-β	M	CTCCCGTGGCTTCTAGTGC	GCCTTAGTTTGGACAGGATCTG
CD86	M	TGTTTCCGTGGAGACGCAAG	TTGAGCCTTTGTAAATGGGCA
NOS2(iNOS)	M	GTTCTCAGCCCAACAATACAAGA	GTGGACGGGTCGATGTCAC
MRC1(CD206)	M	CTCTGTTCAGCTATTGGACGC	CGGAATTTCTGGGATTCAGCTTC
Arg-1	M	CTCCAAGCCAAAGTCCTTAGAG	AGGAGCTGTCATTAGGGACATC

H:human; M:mouse.

### 2.6. Experimental animals and ethical statement

We obtained healthy male C57BL/6 mice (4–8 weeks old, 18–22 g) from the Animal Experimentation Centre of North Sichuan Medical College and acclimated them for 7 days under standard conditions: (22 ± 2)°C, 50–60% relative humidity, and a 12-h light/dark cycle. They were allowed to consume sterilized food and water freely. After one week of acclimatization, the mice were randomly assigned to groups. After intraperitoneal injection of 2 mg/kg LPS to establish an inflammatory model, the mice were divided into two independent experimental modules for functional validation. Mice received intraperitoneal injections for two consecutive days, divided into the following groups: injection of M0-exos (Control group); injection of M0-exos loaded with a METTL3-overexpressing plasmid (OE-METTL3 group); and injection of M0-exos loaded with SI-METTL3 RNA (SI-METTL3 group), to validate the function of METTL3; injection of M0-exos (Control group), injection of M0-exos loaded with miR-155-mimic (miR-155-mimic group), and injection of M0-exos loaded with miR-155-inhibitor (miR-155-inhibitor group), to validate the function of miR-155-5p [41]. forty-eight hours after the final injection, the mice were euthanized by cervical dislocation. Subsequently, under aseptic conditions, the spleen, liver, and lung tissues were harvested using a scalpel, fine scissors, and forceps for subsequent analysis. Each group consisted of 3 mice (*n* = 3), with nine mice used per experiment. Nine mice constituted one experimental unit, and a total of 81 mice were used in this study. Inclusion criteria were: (1) healthy appearance with no visible infection or behavioural abnormalities; (2) body weight within the specified range; (3) stable condition after 7-day acclimatization. Exclusion criteria were: (1) death or severe complications during modelling; (2) failed sample collection or tissue degradation. No animals were excluded from this study. All animal experimental protocols were approved by the Institutional Animal Care and Use Committee of North Sichuan Medical College (NSMC-Ethical Animal Review-2021-10).

### 2.7. GW4869 experiment to inhibit endogenous exosomes

To verify the efficacy and specificity of exosome delivery, mice were randomly divided into two groups: the Exos group and the GW4869 + Exos group. In the GW4869 + Exos group, mice were intraperitoneally pre-treated with GW4869 (2.5 μg/g body weight; dissolved in DMSO; purchased from Yuanye Bio, China) to inhibit endogenous exosome production. One hour later, both groups were simultaneously injected with LPS to induce an acute inflammation model. Three hours after LPS injection, M0-exos loaded with METTL3-overexpressing plasmid were administered. Subsequent tissue collection and detection were performed thereafter.

### 2.8. Flow cytometry (FCM) analysis

Cells (THP-1 or M1 macrophages) were harvested and centrifuged at 1 000 rpm for 5 min. After two washes with PBS, cells were resuspended at a concentration of 2–3 × 10^5^ cells/mL. A 200 μL aliquot of cell suspension was incubated with the following primary antibodies for 30 min: HLA-DR Rabbit mAb (ZenBio, 1:200), Mouse Anti-Human CD86 mAb (ZenBio, 1:500), FITC Anti-Human CD206 (Proteintech, 1:500), FITC Plus Anti-Human CD163 (Proteintech, 1:500). For HLA-DR detection, cells were incubated with FITC-conjugated goat anti-rabbit IgG (ZenBio, at 1:1000) for 45 min. After a final wash, cells were resuspended in 400 μL PBS, filtered through a 100-μm nylon membrane (Labgic, China), and analysed using a flow cytometer (Agilent, China). The mean fluorescence intensity (MFI) was analysed using NovoExpress software (Agilent, China).

### 2.9. Immunohistochemistry (IHC)

Fresh tissue samples were fixed in 4% paraformaldehyde (Dowobio, China) for 18–24 h. After rinsing with running water, gradient ethanol dehydration, transparency, and paraffin embedding, the tissues were sliced into 4–5 µm thick sections. After slicing at 40 °C and baking at 60 °C for 2 h, dewaxing, hydration, and antigen repair were performed. Subsequently, sections were treated with 3% H_2_O_2_ in the dark for 25 min and were then sealed at room temperature with 3% BSA (Servicebio, China) for 30 min. Primary antibodies at appropriate dilutions were applied and incubated overnight at 4 °C. The following day, sections were incubated with an HRP-labelled goat anti-rabbit secondary antibody (Servicebio, 1:200) at room temperature for 50 min. Colour development was performed using DAB substrated kit (Servicebio, China), and cell nuclei were counterstained with haematoxylin. After drying, mount the slide with cover slip adhesive and examine the results under a white light microscope. Use ImageJ software (MediaCybernetics, USA) to quantitatively analyse positive stained areas and measure their average pixel intensity. Primary antibodies and working dilutions are listed below: CD86 (Servicebio, 1:200), iNOS (Servicebio, 1:500), CD206 (Servicebio, 1:400), CD163 (Servicebio, 1:500).

### 2.10. RT-qPCR detection of mRNA expression

Total RNA was extracted from cells or tissues using TRIzol reagent (Thermo, USA). After quantification, the samples were normalized. RNA was reverse transcribed into cDNA using a commercial kit (Thermo, USA) (Reaction system 1: 12 μL; Conditions: 65 °C for 5 min; Reaction system 2: 20 μL; Conditions: 25 °C for 5 min, 42 °C for 60 min, 70 °C for 5 min). Quantitative PCR (qPCR) was performed using a SYBR Green-based kit (ComWin, China) with 1 μL of cDNA template in a 10 μL reaction volume. The reaction protocol was as follows: 95 °C for 5 min (1 cycle); 95 °C for 10 s and 60 °C for 45 s (39 cycles); 95 °C for 15 s, 60 °C for 1 min, 95 °C for 15 s, and 60 °C for 15 s. Relative expression of the target gene was determined using the 2^-ΔΔCt^ method. All primer sequences (Sangon Bio, China) are listed in Table 1.

### 2.11. Probe-based RT-qPCR detection of miRNA expression

Cells were lysed using QIAzol Lysis Reagent (QIAGEN, USA), and miRNA was extracted using the MiRNeasy Micro assay kit (QIAGEN, USA). Mature miR-155-5p was reverse-transcribed into cDNA using the TaqMan^™^ MicroRNA Reverse Transcription Kit (Thermo, USA) (Reaction system: 15 µL; Conditions: 16 °C for 30 min, 42 °C for 30 min, 85 °C for 5 min). Pri-miR-155 was reverse-transcribed into cDNA using the RevertAid First Strand cDNA Synthesis Kit (Thermo, USA) (Reaction system 1: 12 µL; Conditions: 65 °C for 5 min; Reaction system 2: 20 µL; Conditions: 25 °C for 5 min, 42 °C for 60 min, 70 °C for 5 min). QPCR was performed using a TaqMan miRNA assay kit (Gezhi, China) with 2 µL of cDNA product in a 10 µL reaction volume. The reaction protocol was as follows: 95 °C for 2 min; 95 °C for 5 s; 60 °C for 10 s (45 cycles). Using U6 snRNA as an internal reference, the relative expression levels of mature miR-155-5p and pri-miR-155 were determined by the 2^-ΔΔCt^ method. The sequences (Gezhi, China) are listed in Table 1.

### 2.12. m6A dot blot assay

RNA samples were diluted to three concentration gradients of 200, 100, and 50 ng/μL, denatured at 95 °C for 5 min, and immediately subjected to an ice bath for 5 min. 2 μL of each diluted RNA sample was spotted onto an NC membrane and dried for 10 min. RNA was crosslinked to the membrane using UV light for 2 h. Following 1 h blocking in 5% skim milk at room temperature, the membrane was probed with anti-m6A primary antibody (ZenBio, 1:1000) overnight at 4 °C. The following day, the membrane was incubated with HRP-conjugated goat anti-rabbit IgG antibody (ZenBio, 1:5000) at room temperature for 1 h. Signal detection was performed using an enhanced chemiluminescence (ECL) detection system.

### 2.13. Western blot (WB) analysis

Cells or tissue samples were lysed on ice for 30 min using RIPA lysis buffer (Beyotime, China) supplemented with 1% PMSF. When necessary, the lysates were subjected to ultrasonication. The supernatant was collected after centrifugation at 4 °C and 12 000 *g* for 30 min, and the protein concentration was determined using a BCA assay kit (Solarbio, China). Equal amounts of protein were mixed with loading buffer, denatured by heating at 100 °C for 5 min, and separated by SDS-PAGE. The separated proteins were subsequently transferred onto a 0.45-μm PVDF membrane (Millipore, USA). Following a 90-min block in 5% skim milk at room temperature, the membrane was incubated with the appropriate primary antibody overnight at 4 °C. Then, the membrane was incubated with an HRP-labelled goat anti-rabbit IgG antibody (ZenBio, 1:5000) at room temperature for 1 h. Protein bands were detected with an ECL system. Band intensity was quantified by analysing the grayscale values using ImageJ software, and the relative expression of the target protein was normalized to that of the reference protein. Primary antibodies and working dilutions are detailed below: Alix (ABclonal, 1:1000), CD63 (ABclonal, 1:1000), CD9 (ABclonal, 1:1000), NLRP3 (ZenBio, 1:1000), TLR4 (ZenBio, 1:1000), METTL3 (ZenBio, 1:1000), Anti-CD86 (ZenBio, 1:1000), GAPDH (ZenBio, 1:5000), β-tubulin (ZenBio, 1:5000), caspase-1 (HuaAn Bio, 1:500), CD206 (HuaAn Bio, 1:1000), iNOS (Proteintech, 1:1000), Arg-1 (CeneTex, 1:1000).

### 2.14. Enzyme-linked immunosorbent assay (ELISA)

The concentrations of IL-1β, IL-6, and TNF-α in cell culture supernatants and mouse serum were quantified using commercial ELISA kits (NeoBio, China) according to the manufacturer’s protocol. Following preliminary optimisation of sample dilution ratios, 100 μL of standard solution or diluted sample was added to the reaction wells and incubated at 37 °C for 90 min. Following washing, biotinylated antibodies were added and incubated for 60 min, followed by a further wash. The enzyme conjugate was then added and incubated for 30 min. After a final wash, TMB substrate was added and incubated for 15 min. The reaction was terminated with stopping solution, and absorbance was measured at 450 nm using a microplate reader (Thermo, USA).

### 2.15. Intracellular NO detection by DAF-FM DA fluorescence

THP-1 cells were seeded into 96-well plates at 5 × 10^4^ cells/well. After induction, transfection, and the indicated treatments, the culture medium was removed, and cells were loaded with 5 μM DAF-FM DA (Beyotime Bio, China) for 45 min at 37 °C in the dark. Following three washes with PBS, fluorescence was measured at 495/515 nm (excitation/emission) using a microplate reader.

### 2.16. RNA sequencing and data analysis

Total RNA was extracted from the Control and OE-METTL3 groups for transcriptome sequencing. Differentially expressed genes were identified using DESeq2 (|log_2_FC| > 1, adjusted *p* < 0.05), and DO, KEGG, and Reactome enrichment analyses were performed using clusterProfiler (adjusted *p* < 0.05).

### 2.17. Statistical analysis

Results represent at least three independent experiments and are expressed as mean ± SD. Data visualization was performed using GraphPad Prism (GraphPad, USA). Differences between two groups were assessed using a paired t-test, while differences among multiple groups were evaluated using one-way analysis of variance (ANOVA). Differences were considered statistically significant at a *P* value less than 0.05 (*p* < 0.05).

## 3. Results

### 3.1. Exosome identification

**Figure 1.**
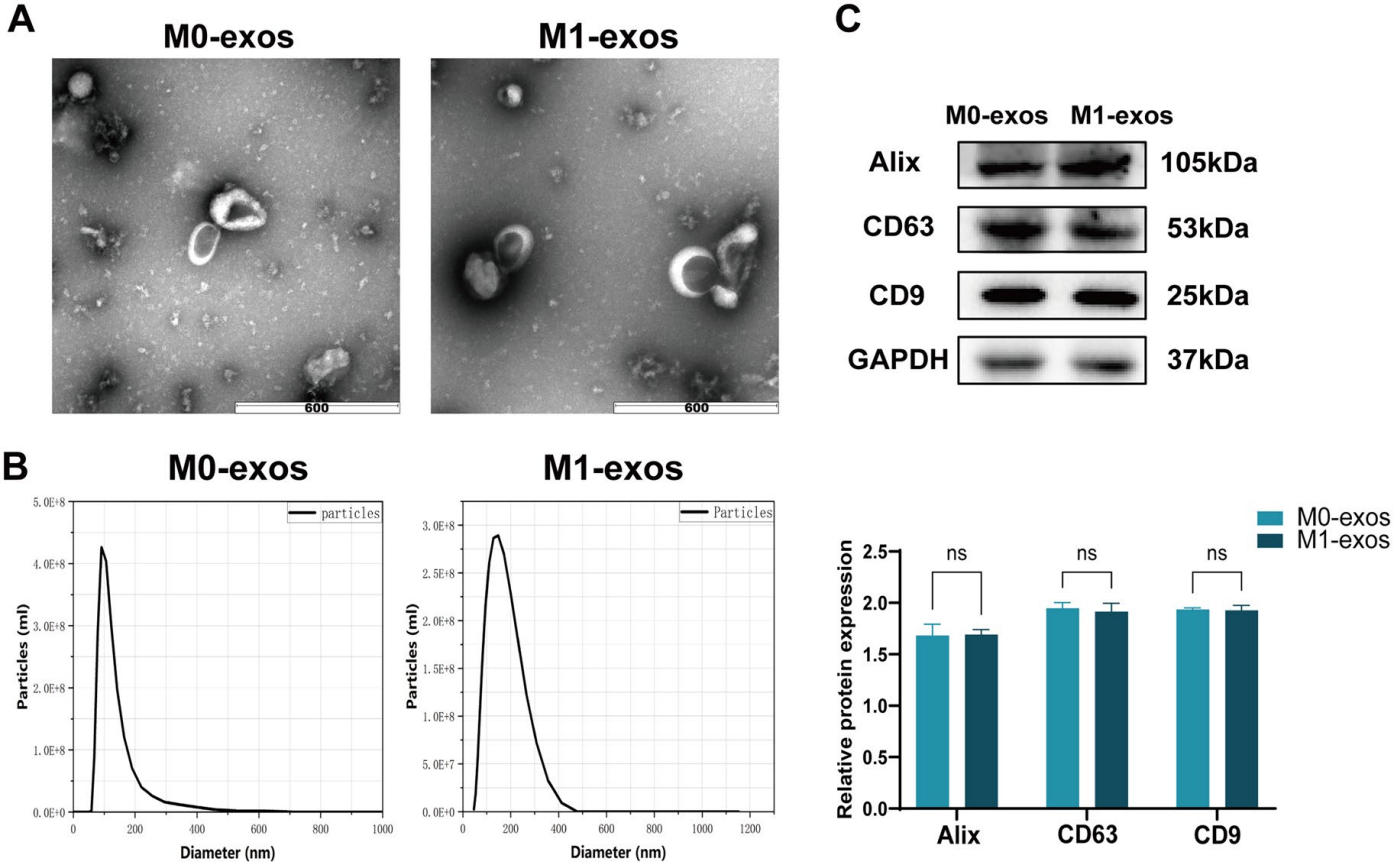
Exosome identification. (A) Exosome morphology and structure. Scale bar, 600 nm. (B) Exosome particle size and concentration. (C) Protein levels of Alix, CD63, and CD9. *ns: p* > 0.05.

Exosomes derived from M0 macrophages (M0-exos) and M1 macrophages (M1-exos) were isolated by ultracentrifugation and characterized. Transmission electron microscopy (TEM) revealed that both exosome populations exhibited typical cup-shaped morphologies (Figure 1(A)). Dynamic light scattering (DLS) showed that 98.4% of M0-exos were distributed within 30–200 nm, with a peak size of 91.3 nm and a concentration of 4.26 × 10^8^ particles/mL; 98.2% of M1-exos were distributed within the same range, with a peak size of 147.7 nm and a concentration of 2.89 × 10^8^ particles/mL (Figure 1(B)), both consistent with exosomal size characteristics. Western blot (WB) analysis confirmed positive expression of exosomal marker proteins Alix, CD63, and CD9 (Figure 1(C)). The above results confirm the successful isolation of exosomes.

### 3.2. Exosomes promote polarization of M1 macrophages by delivering METTL3-mediated m6A modification

#### 3.2.1. Exosomes carrying METTL3 enhance m6A modification to promote the expression of M1 macrophage phenotypic markers

**Figure 2.**
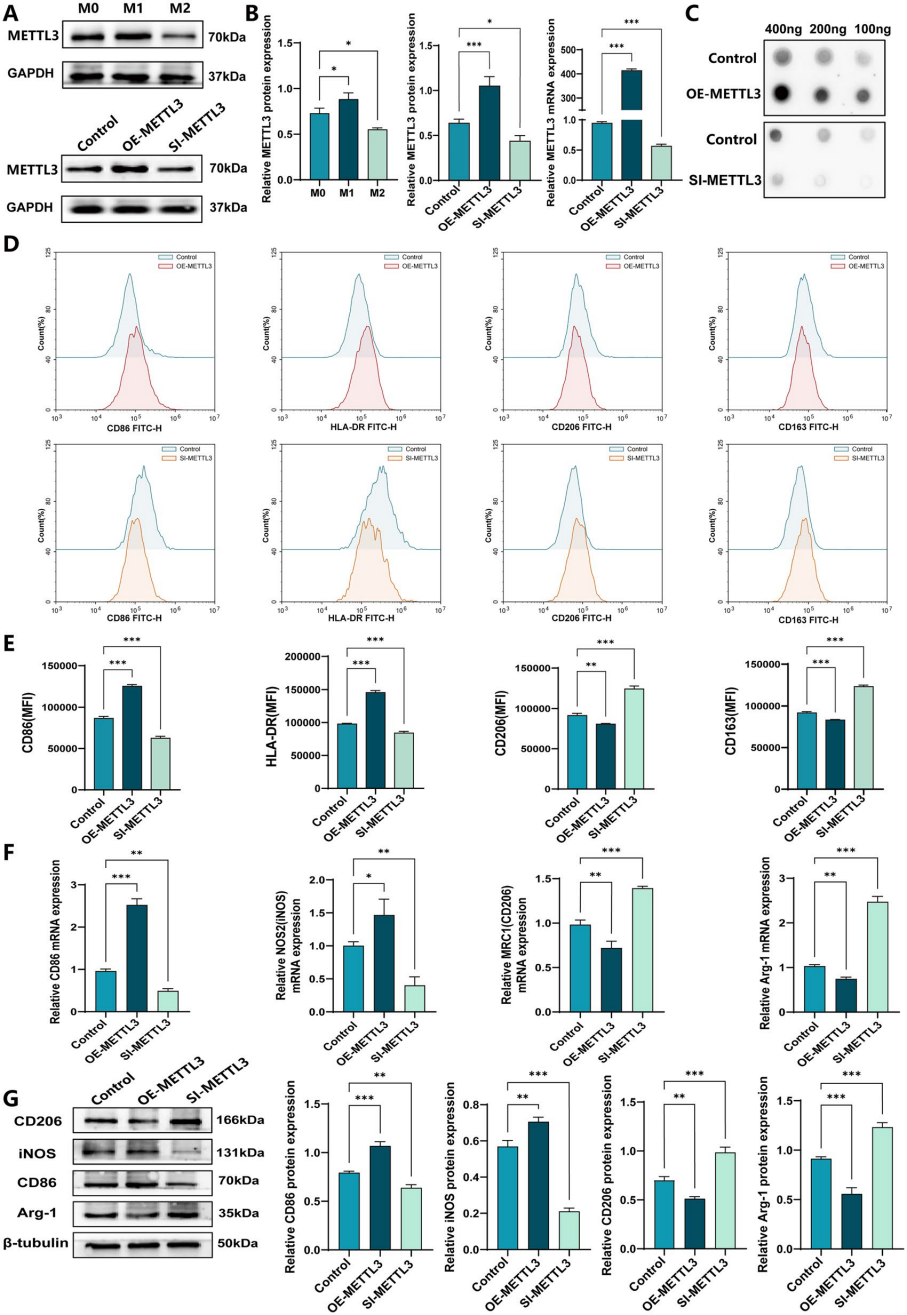
Exosomes carrying METTL3 enhance m6A modification to promote the expression of M1 macrophage phenotypic markers. Control group: M0 macrophages treated with M0-exos; OE-METTL3 group: M0 macrophages treated with M0-exos loaded with METTL3-overexpressing plasmid; SI-METTL3 group: M0 macrophages treated with M0-exos loaded with SI-METTL3 RNA. Subsequently, the three groups were simultaneously induced into M1 macrophages. (A-B) Protein and mRNA expression levels of METTL3 were analyzed by Western blot and RT-qPCR, respectively. (C) The level of m6A modification was detected by Dot blot. (D-E) The expression peaks of CD86, HLA-DR, CD206, and CD163 were detected by FCM, and the MFI results were displayed in a bar chart. (F) The mRNA elaboration levels of CD86, iNOS, CD206 and Arg-1 in cells were detected by RT-qPCR. (G)The protein and mRNA expression levels of CD86, iNOS, CD206 and Arg-1 were quantified by Western blot. *p < 0.05, **p < 0.01, ***p < 0.001.

We first examined METTL3 levels in M0, M1, and M2 macrophages by Western blot. The results showed that METTL3 expression in M0 macrophages was intermediate between that in M1 and M2 cells, representing a baseline level (*p* < 0.05, Figure 2(A–B)). Therefore, selecting M0-exos as the delivery vehicle minimizes interference from pre-existing polarization-related factors. M0-exos were loaded with METTL3 overexpression plasmid or SI-METTL3 RNA, respectively, and then co-incubated with M0 macrophages, with unmodified M0-exos treatment serving as the control group. Subsequently, LPS and IFN-γ were added to all three groups to induce M1 macrophage polarization. Western blot and RT-qPCR analyses revealed that METTL3 protein and mRNA levels were markedly up-regulated or down-regulated relative to the control group (*p* < 0.05, Figure 2(A–B)), and the overall m6A modification level was correspondingly increased or decreased (Figure 2(C)). Flow cytometry (FCM) showed that the mean fluorescence intensity (MFI) of M1 surface markers CD86 and HLA-DR was increased by 1.45- and 1.49-fold in the overexpression group, and decreased by 28% and 14% in the knockdown group, respectively. In contrast, the MFI of M2 markers CD206 and CD163 decreased by 12% and 9% after METTL3 overexpression, and increased by 1.36- and 1.34-fold after knockdown (*p* < 0.01, Figure 2(D–E)). RT-qPCR and Western blot analyses further showed that mRNA expression of CD86 and iNOS was upregulated by 2.63- and 1.46-fold in the OE-METTL3 group, whereas downregulated by 49% and 60% in the SI-METTL3 group; their protein levels were upregulated by 1.35- and 1.24-fold, or downregulated by 19% and 63%, respectively. Additionally, mRNA expression of CD206 and Arg-1 was downregulated by 27% and 28% in the OE-METTL3 group, whereas upregulated by 1.41- and 2.40-fold in the SI-METTL3 group; their protein levels were downregulated by 27% and 39%, or upregulated by 1.40- and 1.35-fold, respectively (*p* < 0.05, Figure 2(F–G)). In summary, these results demonstrate that M0-exos enhance m6A modification levels by delivering METTL3, thereby promoting the expression of M1 phenotypic markers CD86, HLA-DR, and iNOS to drive M1 polarization. Concurrently, this process suppresses the expression of M2 phenotypic markers CD206, CD163, and Arg-1, thereby inhibiting M2 polarization.

#### 3.2.2. Exosomal carrying of METTL3 activates pro-inflammatory signalling pathways to drive macrophage polarization toward the M1 phenotype

**Figure 3.**
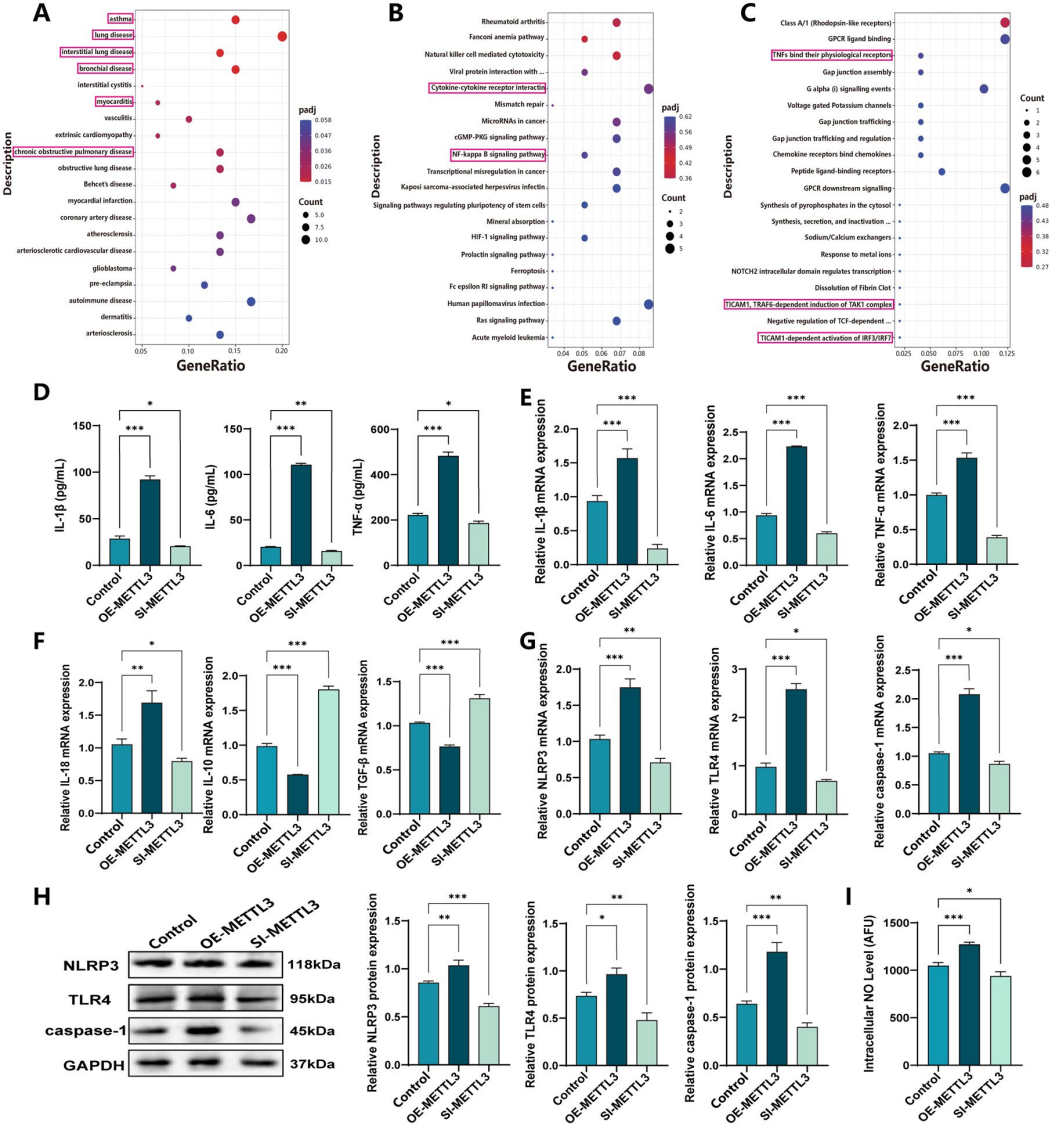
Exosomal carrying of METTL3 activates pro-inflammatory signaling pathways to drive macrophage polarization toward the M1 phenotype. (A–C) Enrichment analysis of differentially expressed genes in the OE-METTL3 group compared with the Control group, including Disease Ontology (DO) enrichment analysis, KEGG pathway enrichment analysis, and Reactome pathway enrichment analysis, respectively. (D) The levels of IL-1β, IL-6, and TNF-α in cell culture supernatants were measured by ELISA. (E–F) The mRNA elaboration levels of IL-1β, IL-6, TNF-α, IL-18, IL-10, and TGF-β in cells were detected by RT-qPCR. (G-H) The mRNA and protein expression levels of NLRP3, TLR4, and caspase-1 were quantified by RT-qPCR and Western blot, respectively. (I) The intracellular NO level was quantified by fluorescence assay. *p < 0.05, **p < 0.01, ***p < 0.001.

To investigate the downstream molecular mechanism by which M0-exos loaded with METTL3 overexpression plasmid induce M1 macrophage polarization, we performed transcriptome sequencing. Disease Ontology (DO) analysis revealed that differentially expressed genes were significantly enriched in inflammatory diseases closely associated with the NLRP3 inflammasome, including asthma, lung disease, myocarditis, and chronic obstructive pulmonary disease (COPD), suggesting that METTL3 may participate in the pathological processes of these diseases through regulation of the NLRP3 inflammasome (*p* < 0.05, Figure 3(A)). KEGG and Reactome pathway analyses further confirmed significant enrichment of inflammatory core pathways, including cytokine-cytokine receptor interaction, NF-κB signaling pathway, and the TLR4 downstream TICAM1/TRAF6/TAK1 complex (*p* > 0.05, Figure 3(B–C)). Subsequently, the levels of inflammatory factors in cell culture supernatants were determined by ELISA. The results showed that the levels of IL-1β, IL-6, and TNF-α were upregulated by 3.23-, 5.47-, and 2.18-fold in the overexpression group, whereas downregulated by 28%, 23%, and 16% in the knockdown group, respectively (*p* < 0.05, Figure 3(D)). The RT-qPCR results showed that the mRNA levels of the pro-inflammatory cytokines IL-1β, IL-6, TNF-α, and IL-18 were increased by 1.68-, 2.39-, 1.54-, and 1.61-fold in the overexpression group, and decreased by 75%, 36%, 61%, and 25% in the knockdown group, respectively. Conversely, the anti-inflammatory factors IL-10 and TGF-β were decreased by 42% and 26% following METTL3 overexpression, and increased by 1.82- and 1.27-fold following knockdown (*p* < 0.05, Figure 3(E–F)). RT-qPCR and Western blot further confirmed that the mRNA expression levels of the inflammation signalling molecules NLRP3, TLR4, and caspase-1 were increased by 1.70-, 2.65-, and 1.98-fold in the overexpression group, and decreased by 37%, 26%, and 19% in the knockdown group. Their corresponding protein levels increased by 1.21-, 1.31-, and 1.85-fold or decreased by 29%, 35%, and 38%, respectively (*p* < 0.05, Figure 3(G–H)). The NO fluorescence intensity levels were upregulated by 1.21-fold in the overexpression group and downregulated by 10% in the knockdown group, respectively (*p* < 0.05, Figure 3(I)). In summary, these results demonstrate that M0-exos enhance m6A modification levels by delivering METTL3, activate the NLRP3/TLR4/caspase-1 signalling pathway, promote the expression of pro-inflammatory factors IL-1β, IL-6, TNF-α, and IL-18, thereby driving M1 polarization; concurrently, they suppress the expression of IL-10 and TGF-β, inhibiting M2 polarization.

#### 3.2.3. Exosomes carrying METTL3 promote the expression of M1 macrophage markers in the mouse spleen

**Figure 4.**
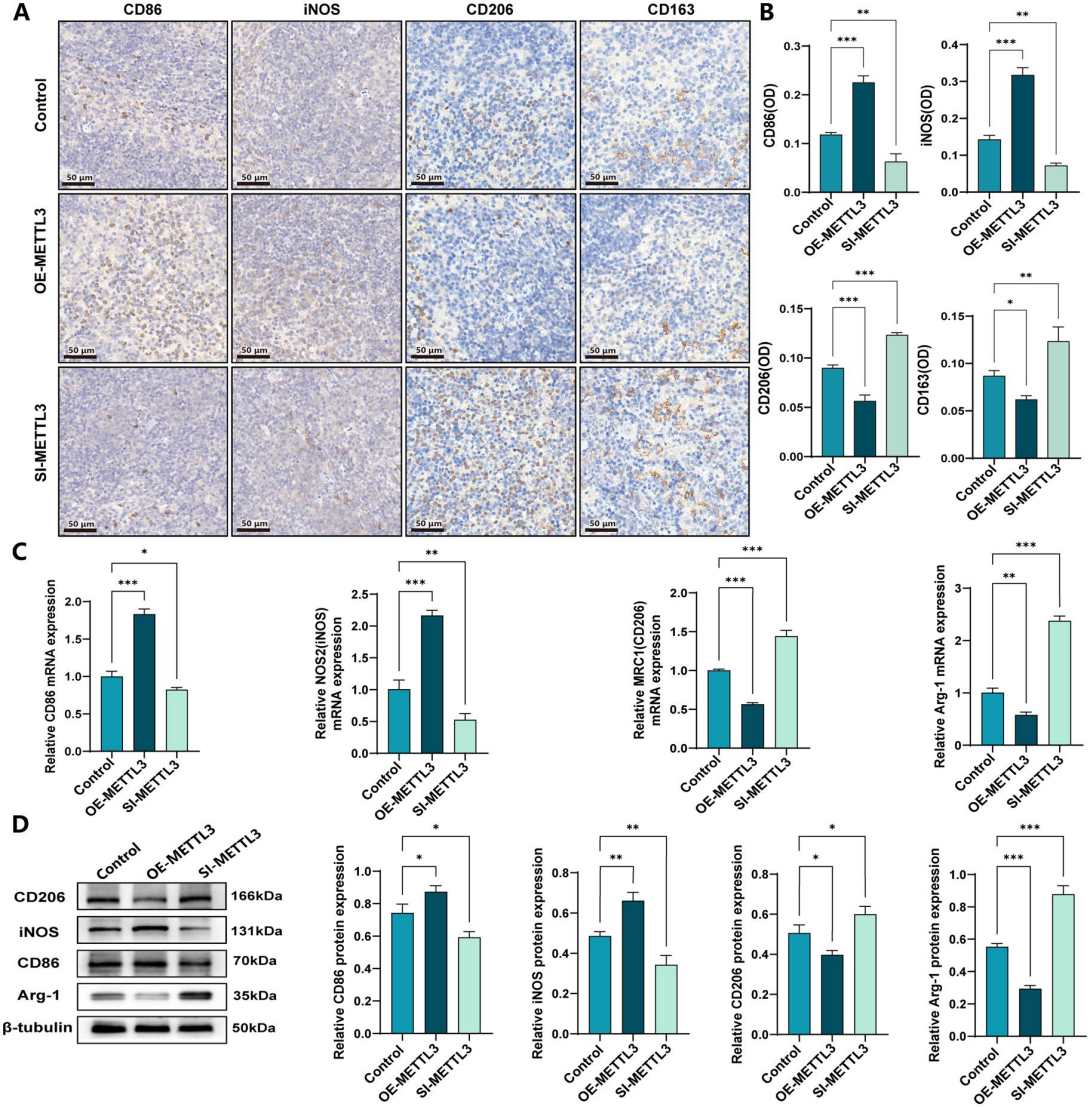
Exosomes carrying METTL3 promote the expression of M1 macrophage markers in the mouse spleen. (A) Representative immunohistochemical (IHC) staining images of CD86, iNOS (M1 markers), CD206, and CD163 (M2 markers) in mouse spleen tissues (scale bar = 50 μm); brown indicates positive staining. (B) Quantification of IHC optical density (OD) values for each marker across experimental groups. (C) The mRNA elaboration levels of CD86, iNOS, CD206, and Arg-1 in mouse spleen tissue were quantified by RT-qPCR. (D) The protein expression levels of CD86, iNOS, CD206, and Arg-1 were quantified by Western blot. *p < 0.05, **p < 0.01, ***p < 0.001.

We further validated the role of METTL3 delivered by exosomes using a mouse model. Immunohistochemical (IHC) analysis of spleen tissues showed that the expression of M1 macrophage markers CD86 and iNOS was significantly increased by 1.91-fold and 2.23-fold in the OE-METTL3 group, and decreased by 47% and 50% in the SI-METTL3 group, respectively. Conversely, the expression of M2 markers CD206 and CD163 decreased by 37% and 29% following METTL3 overexpression, and increased by 1.38-fold and 1.42-fold following METTL3 knockdown (*p <* 0.05, Figure 4(A–B)). RT-qPCR and Western blot analyses further showed that mRNA expression of CD86 and iNOS was upregulated by 1.83- and 2.15-fold in the OE-METTL3 group, whereas downregulated by 18% and 48% in the SI-METTL3 group; their protein levels were upregulated by 1.18- and 1.36-fold, or downregulated by 20% and 29%, respectively. Additionally, mRNA expression of CD206 and Arg-1 was downregulated by 43% and 42% in the OE-METTL3 group, whereas upregulated by 1.44- and 2.37-fold in the SI-METTL3 group; their protein levels were downregulated by 22% and 47%, or upregulated by 1.19- and 1.59-fold, respectively (*p* < 0.05, Figure 4(C–D)). The results showed that M0-exos could deliver METTL3 to recipient tissues, promoting the expression of M1 phenotypic markers CD86 and iNOS, while suppressing the expression of M2 phenotypic markers CD206, CD163, and Arg-1 in mouse spleen tissues.

#### 3.2.4. Exosomal carrying of METTL3 activates pro-inflammatory signaling pathways to drive splenic macrophage polarization toward the M1 phenotype in mice

**Figure 5.**
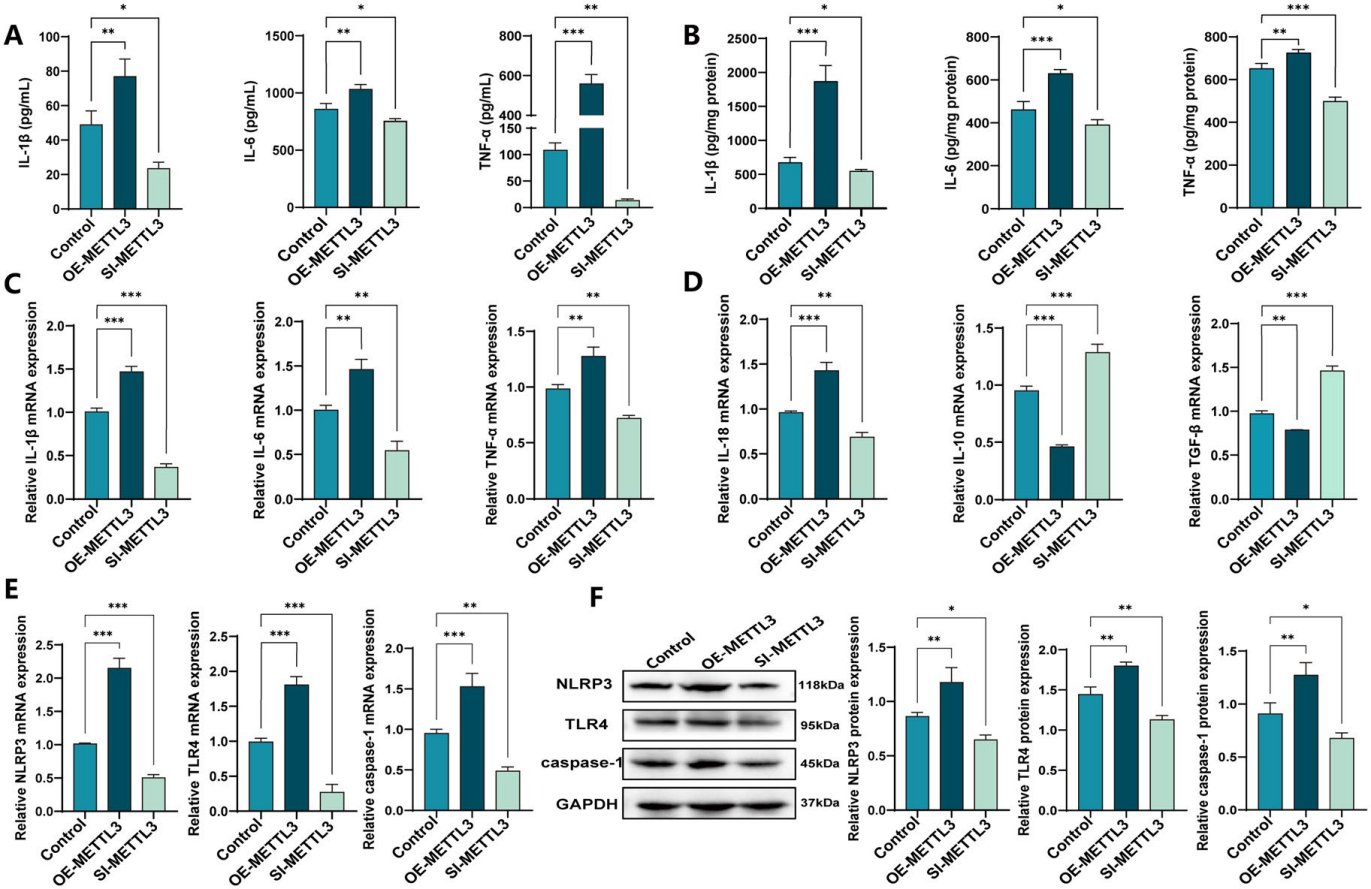
Exosomal carrying of METTL3 activates pro-inflammatory signalling pathways to drive splenic macrophage polarization toward the M1 phenotype in mice. (A–B) The levels of IL-1β, IL-6, and TNF-α in mouse serum and spleen were measured by ELISA. (C–D) The mRNA elaboration levels of IL-1β, IL-6, TNF-α, IL-18, IL-10, and TGF-β in cells were detected by RT-qPCR. (E–F) The mRNA and protein expression levels of NLRP3, TLR4, and caspase-1 were quantified by RT-qPCR and Western blot, respectively. *p < 0.05, **p < 0.01, ***p < 0.001.

To further evaluate the effects of METTL3 overexpression or knockdown on the inflammatory response *in vivo*, we systematically examined the expression levels of related inflammatory factors in mouse serum, spleen tissues, and spleen. ELISA results showed that the levels of IL-1β, IL-6, and TNF-α in mouse serum were upregulated by 1.57-, 1.21-, and 5.15-fold in the overexpression group, whereas downregulated by 52%, 12%, and 87% in the knockdown group, respectively (*p* < 0.05, Figure 5(A)). Similarly, in mouse spleen tissues, the levels of these three inflammatory cytokines were also upregulated by 2.76-, 1.36-, and 1.11-fold in the overexpression group, and downregulated by 19%, 15%, and 23% in the knockdown group, respectively (*p <* 0.05, Figure 5(B)). RT-qPCR results showed that the mRNA levels of the pro‑inflammatory cytokines IL-1β, IL-6, TNF-α, and IL-18 were increased by 1.46-, 1.46-, 1.30-, and 1.48-fold in the OE‑METTL3 group, and decreased by 64%, 46%, 27%, and 28% in the SI-METTL3 group, respectively. The anti‑inflammatory cytokines IL-10 and TGF-β were decreased by 52% and 19% after METTL3 overexpression, and increased by 1.35-fold and 1.50-fold after knockdown (*p* < 0.01, Figure 5(C–D)). RT-qPCR and Western blot analyses further demonstrated that the mRNA expression levels of NLRP3, TLR4, and caspase-1 were increased by 2.13-, 1.82-, and 1.61-fold in the OE-METTL3 group, and decreased by 50%, 73%, and 49% in the SI‑METTL3 group. Their corresponding protein levels were increased by 1.36-, 1.25-, and 1.41-fold or decreased by 25%, 22%, and 25%, respectively (*p* < 0.05, Figure 5(E–F)). These *in vivo* results indicate that M0-exos loaded with METTL3 plasmids, upon injection into mice, can deliver METTL3 to the spleen and drive macrophage polarization toward the M1 phenotype, as evidenced by increased expression of pro‑inflammatory cytokines IL-1β, IL-6, TNF-α, and IL-18, and enhanced activation of the NLRP3/TLR4/caspase-1 signalling pathway. At the same time, METTL3‑enriched exosomes suppress the expression of anti‑inflammatory cytokines IL‑10, TGF‑β, thereby inhibiting M2 polarization.

### 3.3. Exosomal carrying of METTL3 exhibits high efficiency and specificity

**Figure 6.**
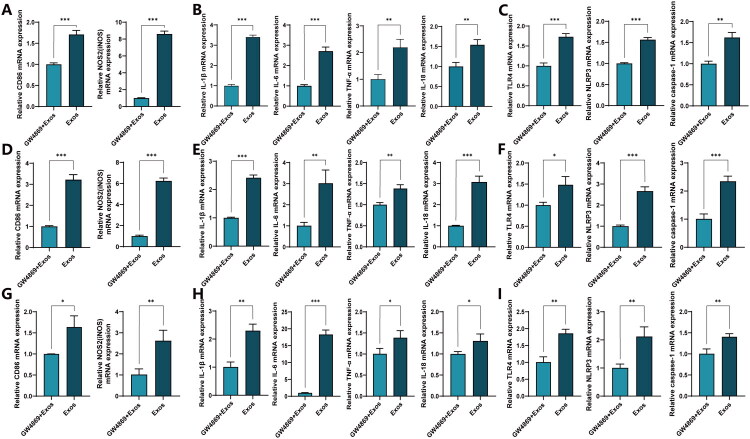
Exosomal carrying of METTL3 exhibits high efficiency and specificity. (A–I) The mRNA expression levels of CD86, iNOS, IL-1β, IL-6, TNF-α, IL-18, TLR4, NLRP3 and caspase-1 in mouse tissues were quantified by RT-qPCR. *p < 0.05, **p < 0.01, ***p < 0.001.

To verify the efficacy and specificity of exosome delivery, we examined the expression levels of phenotypic markers and cytokines in the tissues of mice from the Exos and GW4869 + Exos groups. RT-qPCR analysis of mouse spleen tissues revealed that, compared with the GW4869 group, the mRNA levels of M1 phenotype markers CD86 and iNOS in the Exos group were significantly increased by 1.71-fold and 8.59-fold, respectively (*p* < 0.001, Figure 6(A)); M1-related downstream cytokines IL-1β, IL-6, TNF-α, and IL-18 were elevated by 3.40-fold, 2.71-fold, 2.18-fold, and 1.54-fold, respectively (*p* < 0.01, Figure 6(B)); and signalling pathway molecules TLR4, NLRP3, and caspase-1 were increased by 1.73-fold, 1.56-fold, and 1.62-fold, respectively (*p* < 0.01, Figure 6(C)). In liver tissues, the mRNA levels of CD86 and iNOS in the Exos group were significantly upregulated by 3.22-fold and 6.22-fold, respectively (*p* < 0.001, Figure 6(D)); IL-1β, IL-6, TNF-α, and IL-18 were elevated by 2.42-fold, 3.00-fold, 1.38-fold, and 3.08-fold, respectively (*p* < 0.01, Figure 6(E)); and TLR4, NLRP3, and caspase-1 were increased by 1.48-fold, 2.66-fold, and 2.32-fold, respectively (*p* < 0.05, Figure 6(F)). In lung tissues, the mRNA levels of CD86 and iNOS in the Exos group were significantly upregulated by 1.64-fold and 2.57-fold, respectively (*p* < 0.05, Figure 6(G)); IL-1β, IL-6, TNF-α, and IL-18 were elevated by 2.28-fold, 19.01-fold, 1.38-fold, and 1.31-fold, respectively (*p* < 0.05, Figure 6(H)); and TLR4, NLRP3, and caspase-1 were increased by 1.85-fold, 2.11-fold, and 1.40-fold, respectively (*p* < 0.01, Figure 6(I)). The results from mouse liver and lung tissues were consistent with those observed in the spleen. In summary, M0-exos loaded with overexpressed METTL3 can be effectively delivered to target tissues and exert biological functions, significantly promoting macrophage polarization towards the M1 phenotype; however, these effects were markedly attenuated after GW4869 pre-treatment to block endogenous exosome production, further confirming the efficacy and specificity of METTL3 delivery *via* exosomes.

### 3.4. Exosomes promote the maturation of miR-155-5p through the METTL3-m6A axis

**Figure 7.**
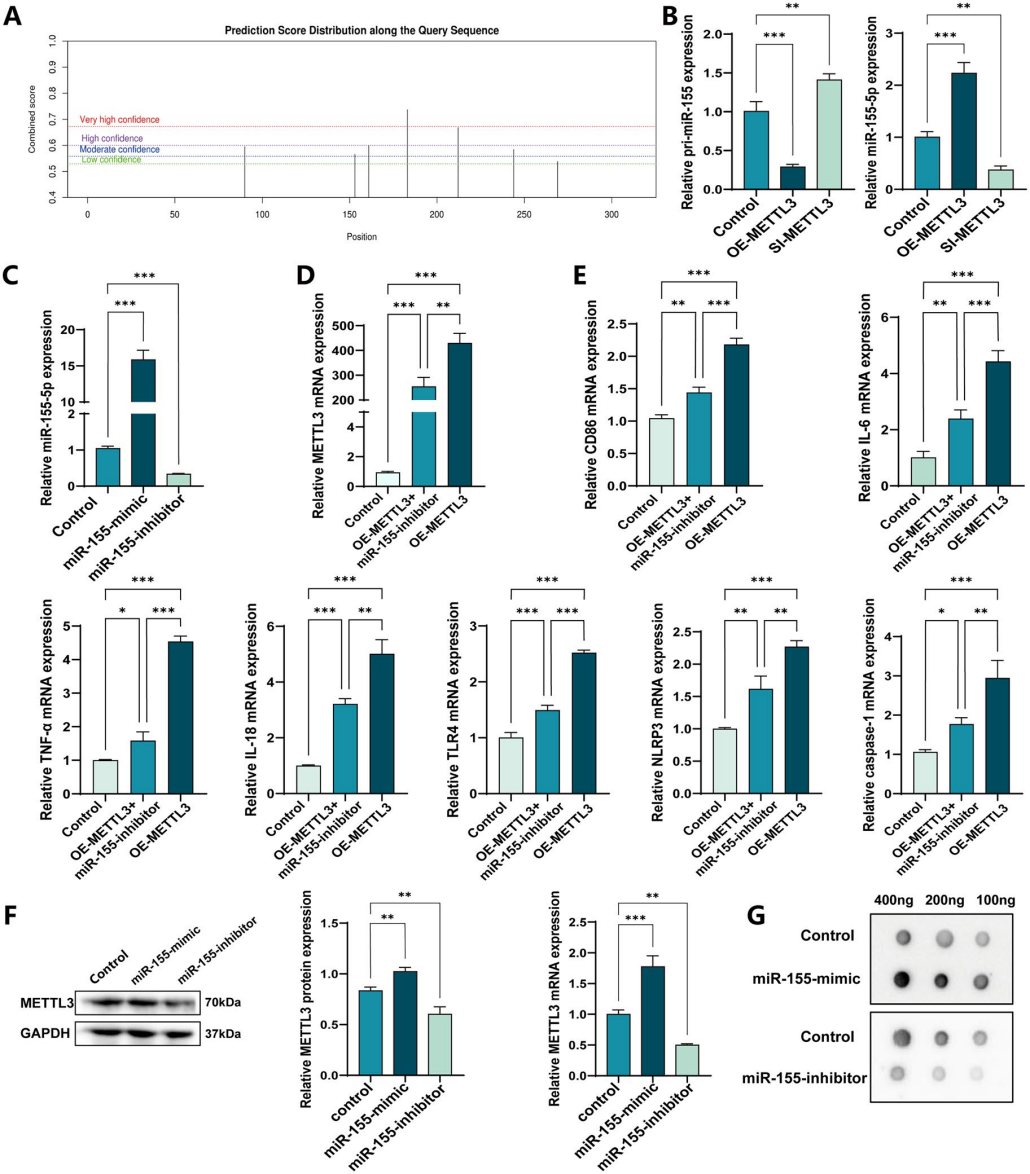
Exosomes promote the maturation of miR-155-5p through the METTL3-m6A axis. Control group: M0 macrophages treated with M0-exos; miR-155-mimic group: M0 macrophages treated with M0-exos loaded with miR-155-mimic; miR-155-inhibitor group: M0 macrophages treated with M0-exos loaded with miR-155-inhibitor; OE-METTL3 + miR-155-inhibitor group: M0 macrophages treated with M0-exos simultaneously loaded with METTL3-overexpressing plasmid and miR-155-inhibitor. Subsequently, these groups were simultaneously induced into M1 macrophages. (A) The m6A modification site on pri-miR-155 was predicted by the SRAMP database. (B–C) RT-qPCR quantified the pri-miR-155 and miR-155-5p levels. (D–E) The mRNA elaboration levels of METTL3, CD86, IL-6, TNF-α, IL-18, TLR4, NLRP3 and caspase-1 in cells were detected by RT-qPCR. (F) The protein and mRNA expression levels of METTL3 were quantified by Western blot and RT-qPCR, respectively. (G) M6A levels were analysed by Dot blot. *p < 0.05, **p < 0.01, ***p < 0.001.

Analysis using the SRAMP database predicted 7 potential m6A methylation sites within pri-miR-155 (Figure 7(A)), indicating a potential role for m6A in governing miR-155 maturation and function. M0-exos were loaded with METTL3 overexpression plasmid or SI-METTL3 RNA, respectively, and then co-incubated with M0 macrophages, and cells were collected 48 h later. RT-qPCR detection results showed that the expression of pri-miR-155 was significantly reduced by 71% in the OE‑METTL3 group and increased by 1.40-fold in the SI‑METTL3 group, while mature miR-155-5p expression was significantly increased by 2.22-fold or decreased by 63%, respectively (*p* < 0.01, Figure 7(B)). Additionally, cells were treated with M0-exos loaded with miR-155 mimics or inhibitors, and the results indicated that the intervention was successful (*p* < 0.001, Figure 7(C)). To investigate whether METTL3 promotes macrophage M1 polarization in a miR-155-5p-dependent manner, M0-exos simultaneously loaded with METTL3 overexpression plasmids and miR-155-5p inhibitors were used to treat macrophages, and downstream cytokines were subsequently detected. RT-qPCR results showed that compared with the OE-METTL3 group, the expression levels of CD86, IL-6, TNF-α, IL-18, TLR4, NLRP3, and caspase-1 in the miR-155-inhibitor group were decreased by 34%, 46%, 65%, 36%, 41%, 29%, and 40%, respectively (*p* < 0.05, Figure 7(D–E)). Western blot and RT-qPCR results showed that the expression level of METTL3 protein was significantly increased by 1.23-fold in the mimic group and decreased by 28% in the inhibitor group (*p* < 0.01, Figure 7(F)). Consistently, METTL3 mRNA expression and m6A modification level were also significantly increased or decreased in the respective groups (Figure 7(G)). These results demonstrate that M0-exos carry METTL3 to recipient macrophages, and the delivered METTL3 subsequently catalyzes m6A modification on pri-miR-155, promoting its maturation into miR-155-5p in the recipient cells. The mature miR-155-5p then drives M1-type polarization. Furthermore, these findings suggest that miR-155-5p serves as a critical downstream effector of METTL3 in driving macrophage M1 polarization.

### 3.5. Exosomes carrying mature miR-155-5p promote the expression of M1 macrophage phenotypic markers

**Figure 8.**
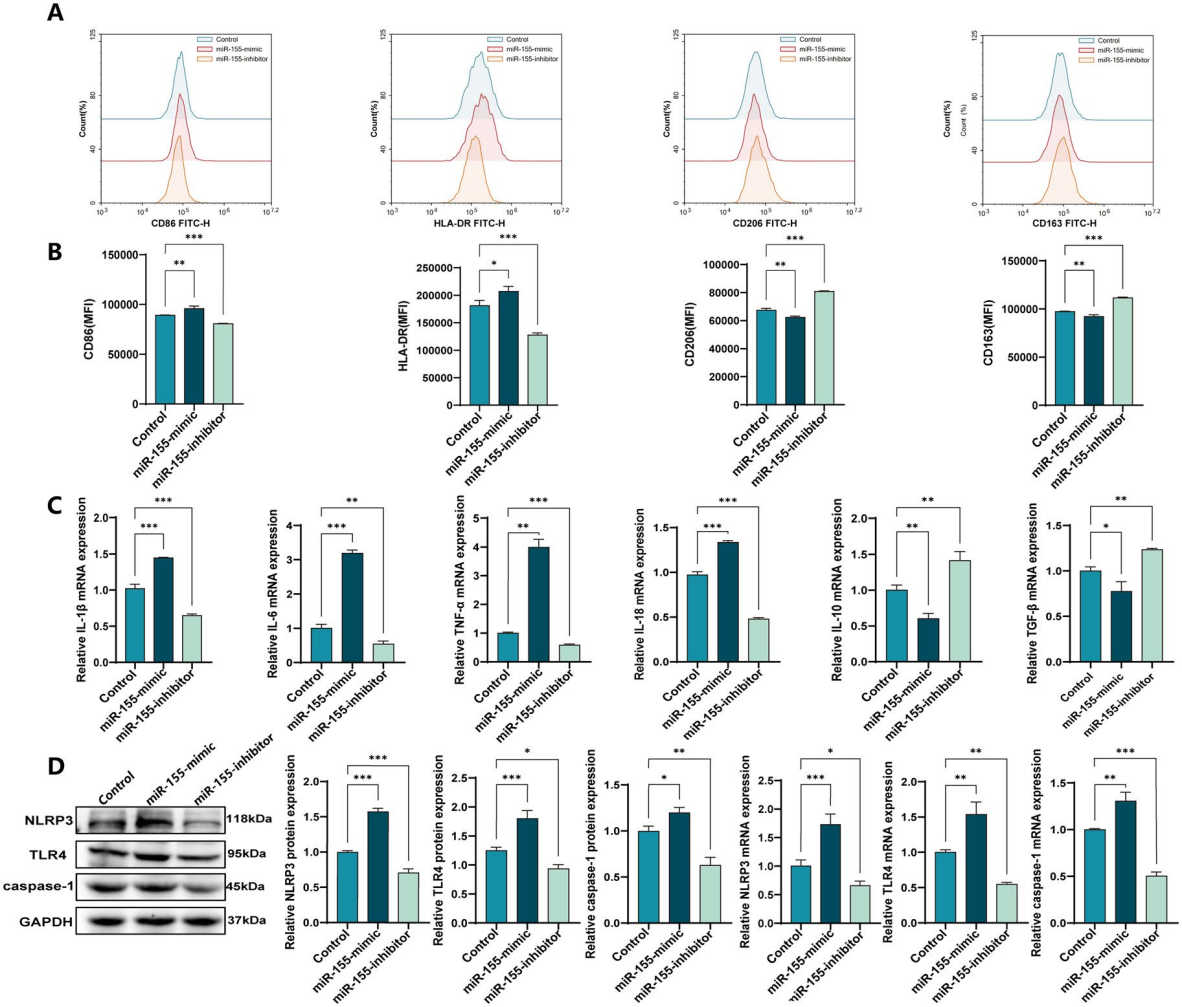
Exosomes carrying mature miR-155-5p promote the expression of M1 macrophage phenotypic markers. (A–B) The expression peaks of CD86, HLA-DR, CD206, and CD163 were detected by FCM, and the MFI values were displayed in a bar chart. (C) RT-qPCR quantified the mRNA levels of IL-1β, IL-6, TNF-α, IL-18, IL-10, and TGF-β. (D) Western blot and RT-qPCR quantified the protein and mRNA levels of NLRP3, TLR4, and caspase-1, respectively. *p < 0.05, **p < 0.01, ***p < 0.001.

We further investigate the direct effect of miR-155-5p delivered by exosomes on macrophage polarization. After loading miR-155-mimic or miR-155 inhibitor into M0-exos and treating the cells, FCM analysis showed that the MFI of M1 surface markers CD86 and HLA-DR increased by 1.07- and 1.14-fold in the mimic group, and decreased by 10% and 30%, in the inhibitor group, respectively. In contrast, the MFI of M2 markers CD206 and CD163 decreased by 7% and 5% following mimic treatment, and increased by 1.20-fold and 1.15-fold following inhibitor treatment (*p* < 0.05, Figure 8(A–B)). RT‑qPCR analysis revealed that the mRNA levels of the pro-inflammatory cytokines IL-1β, IL-6, TNF-α, and IL-18 were increased by 1.42-, 3.17-, 3.99-, and 1.37-fold in the miR‑155‑mimic group, and decreased by 36%, 46%, 41%, and 51% in the inhibitor group, respectively. Conversely, the anti‑inflammatory cytokines IL-10 and TGF-β decreased by 40% and 23% following miR‑155 overexpression, and increased by 1.41-fold and 1.23-fold following its inhibition (*p* < 0.05, Figure 8(C)). Western blot and RT-qPCR analyses further showed that the protein expression levels of NLRP3, TLR4, and caspase-1 were increased by 1.58-, 1.44-, and 1.20-fold in the miR‑155‑mimic group, and decreased by 29%, 25%, and 37% in the inhibitor group, respectively. Their corresponding mRNA levels increased by 1.72-, 1.54-, and 1.31-fold in the mimic group, and decreased by 34%, 45%, and 50% in the inhibitor group (*p* < 0.05, Figure 8(D)). These results demonstrate that exosome-delivered miR-155-5p promotes M1 macrophage polarization by upregulating the expression of surface markers CD86 and HLA-DR, enhancing pro-inflammatory cytokine production IL-1β, IL-6, TNF-α, IL-18, and activating the NLRP3/TLR4/caspase-1 signalling pathway. Additionally, miR-155-5p suppresses the expression of M2 markers CD206, CD163 and anti-inflammatory cytokines IL-10, TGF-β, thereby inhibiting M2 polarization.

### 3.6. Exosomes carrying mature miR-155-5p promote polarization of M1 macrophages in mice

#### 3.6.1. Exosomes carrying mature miR-155-5p promote the expression of M1 macrophage phenotypic markers in mouse spleen

**Figure 9.**
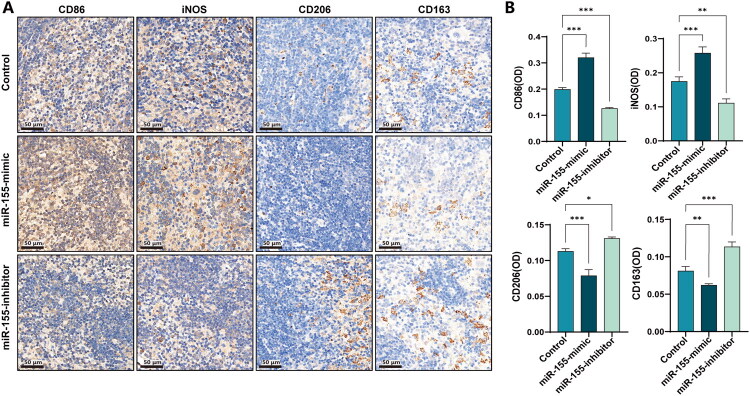
Exosomes carrying mature miR-155-5p promote the expression of M1 macrophage phenotypic markers in mouse spleen. (A) IHC detects CD86, iNOS, CD206 and CD163 indicator images (with a scale of 50 μm), and brown is the positive staining area. (B) Quantification of IHC optical density (OD) values for each marker across experimental groups. *p < 0.05, **p < 0.01, ***p < 0.001.

After loading miR-155-mimic or miR-155-inhibitor into M0-exos and treating mice, IHC results revealed that the expression of M1 markers CD86 and iNOS increased by 1.61-fold and 1.48-fold in the miR‑155‑mimic group, and decreased by 37% and 36% in the inhibitor group, respectively. In contrast, the M2 markers CD206 and CD163 decreased by 30% and 23% after miR‑155 overexpression and increased by 1.16-fold and 1.40-fold following its inhibition (*p* < 0.05, Figure 9(A–B)). These results indicate that exosome-delivered miR-155-5p promotes M1 polarization in mouse splenic macrophages by upregulating CD86 and iNOS, while concurrently suppressing the expression of M2 markers CD206 and CD163.

#### 3.6.2. Exosomal carrying of mature miR-155-5p activates pro-inflammatory signalling pathways to drive macrophage polarization towards the M1 phenotype in vivo

Western blot and RT-qPCR detection results revealed that miR‑155‑5p carried by exosomes enhanced the expression of key inflammatory signalling molecules in a tissue‑specific manner.

**Figure 10.**
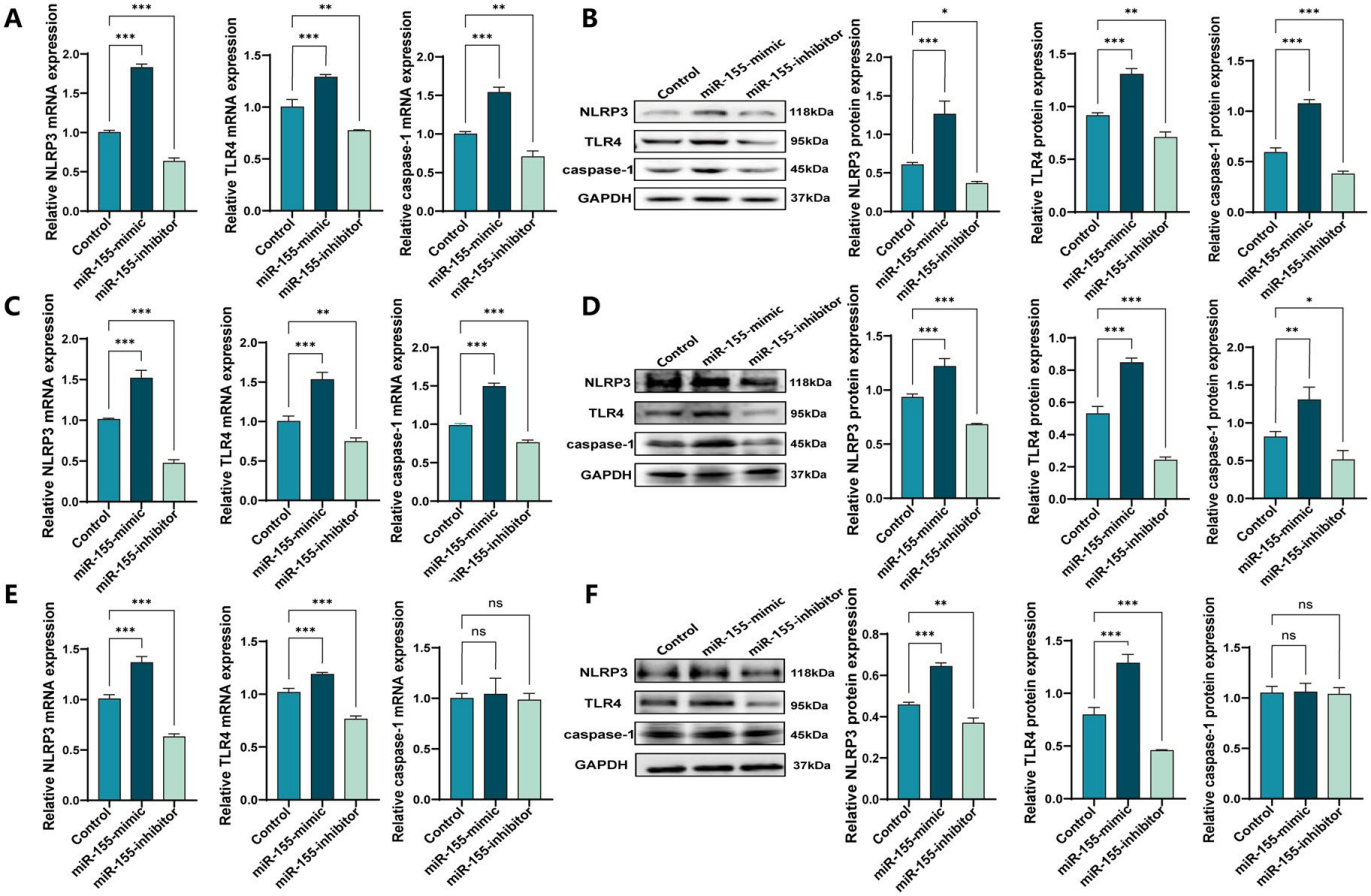
Exosomal carrying of mature miR-155-5p activates pro-inflammatory signalling pathways to drive macrophage polarization towards the M1 phenotype *in vivo*. (A-F) Protein and mRNA levels of NLRP3, TLR4, and caspase-1 in mouse tissues were determined by RT-qPCR and Western blot, respectively. ns:*p* > 0.05, *p < 0.05, **p < 0.01, ***p < 0.001.

In the spleen, mRNA levels of NLRP3, TLR4, and caspase-1 increased by 1.83-, 1.29-, and 1.54-fold in the mimic group, and decreased by 37%, 23%, and 30% in the inhibitor group. Their protein levels were increased by 2.08-, 1.43-, and 1.81-fold or decreased by 40%, 23%, and 36%, respectively (*p* < 0.05, Figure 10(A–B)). In the liver, NLRP3, TLR4, and caspase-1mRNA expression increased by 1.50-, 1.53-, and 1.51-fold with miR‑155 mimic, and decreased by 53%, 26%, and 23%with inhibitor treatment. Corresponding protein levels were increased by 1.30-, 1.60-, and 1.60-fold or decreased by 27%, 54%, and 37% (*p* < 0.05, Figure 10(C–D)). In the lung, protein expression of NLRP3 and TLR4 increased by 1.41-fold and 1.61-fold in the mimic group and decreased by 19% and 42% in the inhibitor group; mRNA levels showed similar trends (*p* < 0.01, Figure 10(E–F)); however, neither the mRNA nor protein levels of caspase-1 in lung tissue exhibited significant differences between groups (*p* > 0.05, Figure 10(E–F)).

Together, these data indicate that exosome-delivered miR-155-5p enhances the NLRP3/TLR4/caspase-1 signalling pathway in the spleen and liver, whereas the lung exhibits a more restricted response, further underscoring the organ-specificity of miR-155-5p-driven macrophage polarization.

#### 3.6.3. Exosomes carrying mature miR-155-5p enhance pro-inflammatory cytokine expression across multiple mouse organs

**Figure 11.**
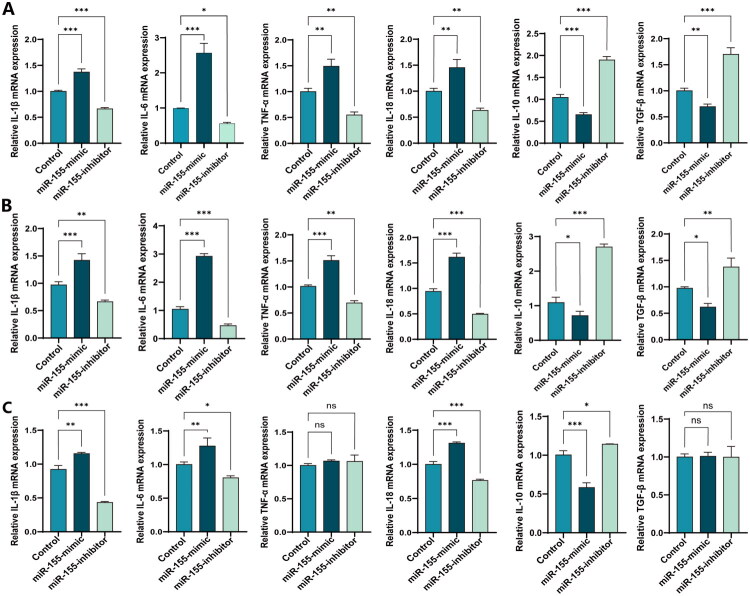
Exosomes carrying mature miR-155-5p enhance pro-inflammatory cytokine expression across multiple mouse organs. (A–C) The mRNA expression levels of IL-1β, IL-6, TNF-α, IL-18, IL-10, and TGF-β in mouse tissues were quantified by RT-qPCR. *p < 0.05, **p < 0.01, ***p < 0.001.

RT-qPCR detection of mouse tissues showed that miR‑155‑5p overexpression in exosomes significantly upregulated mRNA levels of pro‑inflammatory cytokines. In the spleen, IL‑1β, IL‑6, TNF‑α, and IL-18 increased by 1.37-, 2.58-, 1.49-, and 1.38‑fold, respectively, while their expression decreased by 35%-45% in the inhibitor group. Anti‑inflammatory cytokines IL-10 and TGF‑β were decreased by 38% and 30% in the mimic group and increased by 1.82-fold and 1.70-fold, following inhibition (*p* < 0.05, Figure 11(A)). In the liver, IL-1β, IL-6, and IL‑18 increased by 1.46-, 2.92-, 1.49-, and 1.71-fold with miR-155 mimic treatment, and decreased by 31%-54% with inhibitor treatment. IL-10 and TGF‑β were decreased by 35% and 37% in the mimic group and increased by 2.47-fold and 1.41-fold in the inhibitor group (*p* < 0.05, Figure 11(B)). In the lung, IL-1β, IL-6, and IL-18 were increased by 1.25-, 1.35-, and 1.31-fold in the mimic group and decreased by 53%, 20%, and 24% in the inhibitor group (*p* < 0.05, Figure 11(C)). Notably, TNF‑α and TGF‑β levels in lung tissue revealed no statistically significant differences across groups (*p* > 0.05, Figure 11(C)). These findings demonstrate that exosome‑borne miR‑155‑5p enhances pro‑inflammatory cytokine extrusion and inhibits anti‑inflammatory factor secretion in the spleen and liver. The attenuated response in lung tissue suggests organ‑specific modulation of macrophage polarization.

## 4. Discussion

Macrophage polarization represents a central process in immune response and tissue homeostasis, dynamically regulated by transcription factors and epigenetic mechanisms. Beyond classical pathways such as signal transducer and activator of nuclear factor-κB (NF-κB), transcription-1 (STAT-1), and peroxisome proliferator-activated receptors (PPARs), recent studies highlight the key roles of m6A RNA methylation modification and non-coding RNA networks in modulating macrophage polarization [42–44]. METTL3, the pivotal methyltransferase driving m6A deposition, promotes M1 polarization by modifying specific downstream targets [45–46]. In addition, exosomes deliver functional microRNAs (e.g., miR-155-5p) to recipient cells, thereby shaping the immune microenvironment [47–48]. However, whether miR-155-5p in exosomes is regulated by METTL3-mediated m6A modification remains unclear; elucidating this axis holds significant implications for inflammation and tumour immunotherapy.

In this study, we found that exosomes promote M1 macrophages polarization by delivering METTL3 and enhancing m6A modification. These vesicles deliver METTL3 to macrophages, which catalyses m6A methylation of TLR4 mRNA, thereby enhancing its stability and translation efficiency and upregulating TLR4 expression on the cell membrane. This finding aligns with reports by Luo et al. in neutrophils [49]. Enhanced TLR4 signalling elevates transcription levels of NLRP3, pro-IL-1β, pro-IL-18, and pro-caspase-1. NLRP3 then recruits ASC and pro-caspase-1 to form the inflammasome, activating caspase-1. Activated caspase-1 cleaves pro-IL-1β and pro-IL-18 into their mature forms, induces iNOS to produce nitric oxide (NO), and cleaves gasdermin D (GSDMD) to induce pyroptosis—collectively amplifying M1 polarization and pro‑inflammatory responses. At this time, cells upregulate M1 markers CD86 and HLA-DR, enhancing antigen presentation and T cell interaction. During this process, M2 polarization is inhibited, as shown by decreased CD206 and CD163 levels and diminished secretion of IL-10 and TGF-β, indirectly reinforces M1 dominance.

To verify these mechanisms *in vivo*, we constructed an LPS/IFN-γ-induced acute inflammation model in mice. Results confirmed that M0-exos carrying METTL3 drive splenic macrophages toward M1 polarization, enhancing NLRP3, TLR4, caspase-1, pro‑inflammatory cytokines IL-1β, IL-6, TNF-α, and IL-18, and M1 markers CD86, iNOS, while downregulating M2 markers CD206, CD163 and anti‑inflammatory cytokines IL-10, TGF-β. This aligns with Tong et al. [50], who identified METTL3-mediated m6A modification as a key positive regulator of macrophage activation through CRISPR screening technology. Moreover, after GW4869 pretreatment blocked endogenous exosome production, the pro-M1 polarization effect of METTL3-overexpressing exosomes was significantly attenuated, further confirming the specificity and necessity of exosomes as a METTL3 delivery vehicle. Thus, M0-exos enhance M1 polarization in recipient macrophages through METTL3-dependent m6A modification.

It is worth noting that microRNAs are key targets of m6A modification, and miR-155-5p is a well-established driver of M1 polarization [51]. Using the SRAMP database, we identified two high-confidence m6A modification sites within pri-miR-155, suggesting its processing is m6A-regulated. Co-culture of METTL3-overexpressing M0-exos with target cells significantly reduced the abundance of pri-miR-155 while increasing mature miR-155-5p by 2.2-fold; this effect was reversed upon METTL3 knockdown, confirming METTL3-dependent maturation. We established a combined treatment group of METTL3 overexpression with miR-155-5p inhibitor. The results showed that M1 macrophage polarization in this group was significantly attenuated compared with the METTL3 overexpression alone group, indicating that miR-155-5p is a key downstream target mediating the pro-M1 polarization effect of METTL3. M0-exos deliver METTL3 to recipient macrophages, and the delivered METTL3 subsequently catalyses m6A modification on pri-miR-155, promoting its maturation into miR-155-5p in the recipient cells. The possible mechanism is that the generation of microRNAs depends on the cleavage of primary transcripts by the DGCR8/DROSHA complex. The ‘marker’ of m6A on pri-miRNA can be directly read by DGCR8, recruiting DROSHA to complete efficient cleavage. The deletion of METTL3 weakens the binding ability of DGCR8 to precursor microRNAs, suggesting that m6A methylation serves as a critical post-transcriptional mark that can label substrates and promote the initiation of miRNA biosynthesis, as reported by Alarcón et al. [52–53].

To determine whether exosome‑driven M1 polarization depends on METTL3‑mediated miR‑155‑5p maturation, we treated cells with M0-exos transfected with miR-155 mimic or inhibitor. Results showed that exosome-delivered miR-155-5p upregulated M1 markers CD86 and HLA-DR, activated NLRP3/TLR4/caspase-1 signalling, and enhanced secretion of iNOS, TNF-α, IL-1β, IL-6, and IL-18, while concurrently down-regulating the M2 markers CD206 and CD163 together with the anti-inflammatory cytokines IL-10 and TGF-β. A reasonable explanation is that miR-155-5p enhances pro-inflammatory pathways (e.g. NF- κB/STAT1), increasing TLR4 and NLRP3 expression. This drives NLRP3 inflammasome assembly, activates caspase-1, and promotes the maturation of IL-1β and IL-18, which stabilize the M1 phenotype through autocrine/paracrine loops and increase IL-6 and TNF-α production. Simultaneously, miR-155-5p targets the 3′-UTR of IL-13 Rα1, inhibiting STAT6 activation [54], downregulating M2 markers and blunting IL-10/TGF-β secretion.

*In vivo*, we used a mouse inflammation model to administer the same intervention treatment as *in vitro*, and the results of spleen and liver tests were consistent with the *in vitro* trends. Whereas lung tissue showed a significant increase in early factors such as IL-1β and IL-18 but non-significant changes in TNF-α and TGF-β. This organ‑specificity may be due to the dependence of alveolar macrophages on STAT3/IL-10 autocrine loops maintaining high SHIP1/SOCS1 levels [55–56]. MiR-155 may need to degrade these negative regulatory proteins to relieve the inhibition of inflammatory signals, and further induction of TNF-α may be limited by the incomplete clearance of residual SOCS1 activity. Overall, both *in vitro* and *in vivo* results fully demonstrate that M0-exos utilize the METTL3-m6A-miR-155-5p axis to disrupt M1/M2 balance and lock macrophages into an M1-polarized state.

Collectively, our findings position METTL3 not only as an m6A ‘writer’ but also as an exosome-packaged cargo that propagates methylation events across cells, enabling rapid miR‑155‑5p maturation and M1 polarization amplification—providing a new post- transcriptional regulatory mechanism in intercellular communication. We acknowledge the current exosomes mixed with microvesicles due to the limitations of exosome separation technology; however, control groups have been set up to exclude this confounder in our research. Future advances in exosomes subpopulation purification will further refine research in this field.

## 5. Conclusion

**Figure 12.**
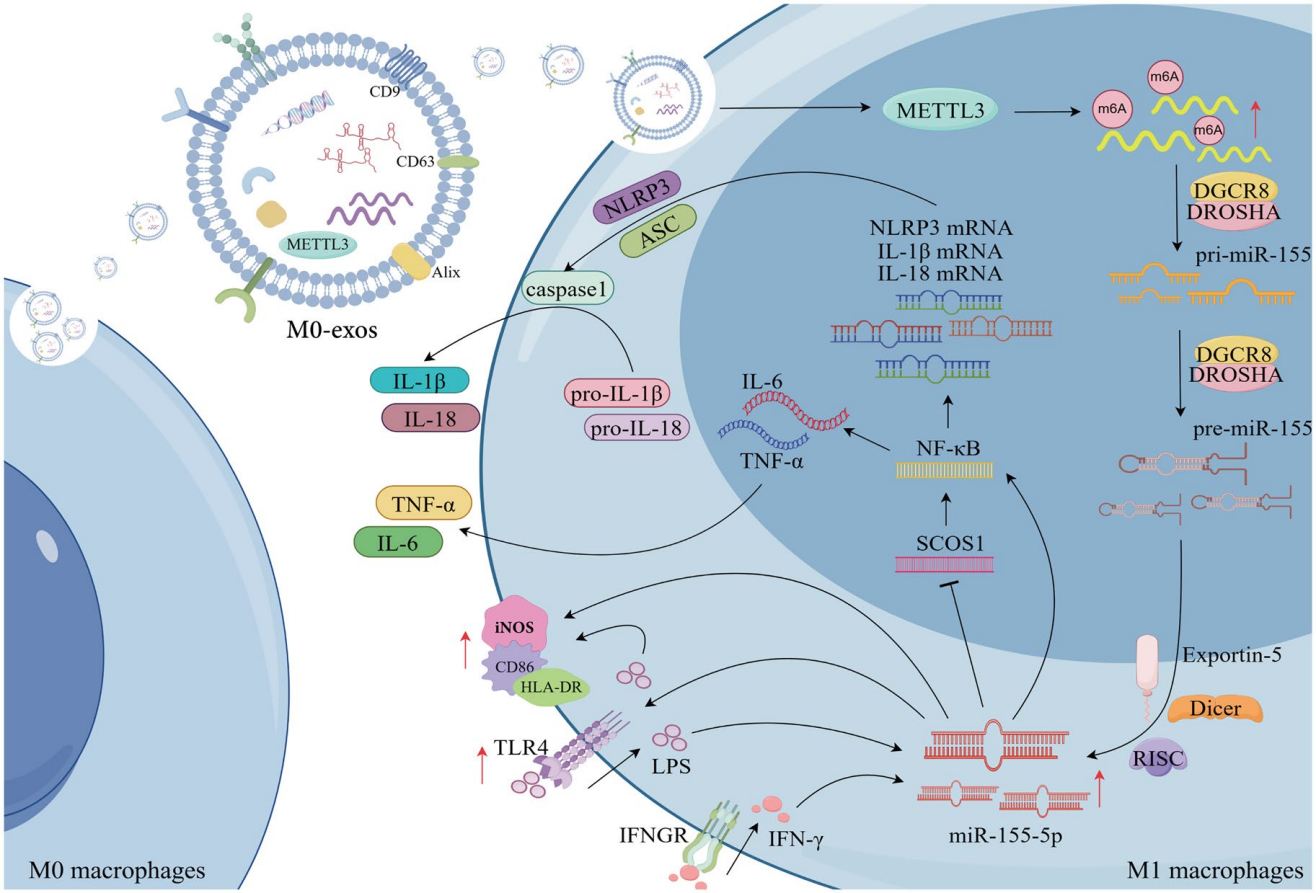
Mechanism diagram of exosomes-mediated m6A modification promoting M1 macrophage polarization through METTL3 regulation of miR-155-5p maturation. M0 macrophage-derived exosomes (M0-exos) loaded with the METTL3 overexpression gene can carry METTL3 to recipient M0 macrophages. The carried METTL3 subsequently catalyzes m6A modification on pri-miR-155, promoting its maturation into miR-155-5p within the recipient macrophages. The mature miR-155-5p activates caspase-1 by upregulating the expression levels of NLRP3 and TLR4, thereby significantly promoting the secretion of inflammatory factors including TNF-α, IL-1β, IL-6, and IL-18. Meanwhile, the expression of M1 macrophage markers such as CD86 and HLA-DR is upregulated, inducing the polarization of M0 macrophages toward the M1 phenotype, which further amplifies the inflammatory response.

In summary, this study established that M0 macrophage-derived exosomes deliver METTL3 to mediate m6A-dependent maturation of miR-155-5p, thereby driving polarization of M1 macrophages. As illustrated in Figure 12, this axis upregulates M1 markers CD86 and HLA-DR, activates the NLRP3/TLR4/caspase-1 signalling pathway, and promotes the release of pro-inflammatory mediators (IL-1β, IL-6, TNF-α, and IL-18), thereby exacerbating inflammatory responses. Our work elucidates the ‘exosomes-METTL3-m6A-miR-155-5p’ regulatory axis and deciphers its associated signalling network, providing new potential targets for inflammation and tumour immunotherapy.

## Data Availability

All data required to evaluate the conclusions are included in the paper and are available from the authors upon request.
